# Action-Effect Associations in Voluntary and Cued Task-Switching

**DOI:** 10.3389/fpsyg.2017.02233

**Published:** 2018-01-17

**Authors:** Angelika Sommer, Sarah Lukas

**Affiliations:** ^1^Pedagogic Psychology, University of Education Weingarten, Weingarten, Germany; ^2^Institute for Research, Development and Evaluation, University of Teacher Education Bern, Bern, Switzerland

**Keywords:** consistency effect, non-reversal advantage, ideomotor action control mode, sensorimotor action control mode, action-effect learning, voluntary task-switching, cued task-switching

## Abstract

The literature of action control claims that humans control their actions in two ways. In the stimulus-based approach, actions are triggered by external stimuli. In the ideomotor approach, actions are elicited endogenously and controlled by the intended goal. In the current study, our purpose was to investigate whether these two action control modes affect task-switching differently. We combined a classical task-switching paradigm with action-effect learning. Both experiments consisted of two experimental phases: an acquisition phase, in which associations between task, response and subsequent action effects were learned and a test phase, in which the effects of these associations were tested on task performance by presenting the former action effects as preceding effects, prior to the task (called *practiced effects*). Subjects either chose freely between tasks (ideomotor action control mode) or they were cued as to which task to perform (sensorimotor action control mode). We aimed to replicate the consistency effect (i.e., task is chosen according to the practiced task-effect association) and non-reversal advantage (i.e., better task performance when the practiced effect matches the previously learned task-effect association). Our results suggest that participants acquired stable action-effect associations independently of the learning mode. The *consistency effect* (Experiment 1) could be shown, independent of the learning mode, but only on the response-level. The *non-reversal advantage* (Experiment 2) was only evident in the error rates and only for participants who had practiced in the ideomotor action control mode.

## Introduction

Human actions are either exogenously or endogenously controlled (e.g., Herwig et al., 2007; Gaschler and Nattkemper, 2012). In the first case, actions are triggered by external stimulation, i.e., crossing a street because the traffic light turns green or preparing a speech because you are invited to give a presentation. In the latter case, actions are performed to achieve a current goal, i.e., crossing a street because the bookshop to which you want to go is on the other side of the street or booking a train ticket to go on holiday to Amalfi. Thus, in accordance with the stimulus-based approach, humans respond to external stimuli in order to accommodate environmental demands.

Ideomotor approaches emphasize that the cognitive representation of action effects plays a crucial role in action planning (Lotze, 1852; James, 1890/1981; Kunde et al., 2007). According to the ideomotor principle, the motor execution of an action is triggered by the anticipation of the expected action effect. The binding link between sensory events and motor movements has been studied extensively. It is assumed that actions are cognitively represented by codes that capture their sensory events (Prinz, 1990, 1997). In several models of action control, e.g., Hommel’s action-concept model, (Hommel, 1993, 1996) or the theory of event coding (Hommel et al., 2001) action features and sensory events are represented in shared feature codes. As pointed out by Elsner and Hommel (2001) bidirectional learning is an essential precondition for intention-based actions. This means that the learning between (motor) action and (sensory) effect may lead to the activation of a motor response when perceiving the sensory event or endogenously activating its representation. In their two phase-model of action control, motor patterns and sensory effects contingently co-occur (first phase) and are consequently integrated in common coding units (second phase). In line with this theory, Elsner and Hommel’s (2001) experiments consisted of two experimental phases: In the acquisition phase, participants pressed a left or a right key with their index fingers either in forced-choice designs (participants were cued as to which key to press) or in free-choice designs (they were allowed to choose which key to press within each trial). Responses were followed by a high or a low-pitched tone depending on the pressed key. In the test phase, the previous action-effects were presented as imperative stimuli *before* task execution. According to the ideomotor principle, presenting these action effects should activate the representation of these actions. In Experiment 1, they employed a forced-choice test phase in which participants had to respond to the action effects either with correspondent or reversed tone-key mapping. Subjects performed better with a non-reversed tone-key mapping when compared to reversed mapping (the *non-reversal advantage*, cf. Pfister et al., 2011). In the following experiments, free-choice test phases were employed. Participants had to randomly choose one of two keys after the previous action-effect was presented. As part of these experiments, subjects selected the key that had produced the presented tone in the acquisition phase. This result pattern is referred to as *consistency effect* (cf. Pfister et al., 2011).

The acquisition and use of learned action-effect associations have been addressed in numerous studies either employing a free-choice test phase (e.g., Hommel et al., 2003; Hoffmann et al., 2009) or a forced-choice test phase (e g., Maes, 2006; Hoffmann et al., 2009). In the acquisition phases participants usually performed free-choices between the two response alternatives. As Herwig et al. (2007) and Herwig and Waszak (2009) pointed out, the learning mode in the acquisition phase may also influence the integration of action-effects in the ensuing test phase. Therefore, they contrasted a free-choice acquisition phase with a forced-choice acquisition phase. By testing the impact of the acquisition phase in a forced-choice test phase, they found a non-reversal advantage for the free choice acquisition group, but not for the forced choice acquisition group. Therefore, Herwig et al. (2007) and Herwig and Waszak (2009), concluded that participants who had undergone stimulus-based learning did not acquire action-effect links. Experiments on stimulus-response compatibility (Kunde, 2003) and stimulus-effect compatibility (Hommel, 1996) suggest another explanation. It is assumed that participants acquire testable action-effect associations in both learning modes, but only the free-choice acquisition group uses the action-effect links in the test phase. To test this alternative explanation, Pfister et al. (2011) performed the same experiment as Herwig et al. (2007) but replaced the forced-choice test phase by a free-choice design. If both acquisition groups (free-choice and forced-choice) learned action-effect associations, participants who acquired action-effect binding in the forced-choice group should also show a consistency effect in a free-choice test phase. This is what the authors could show. Their results indicated that the *acquisition* of action-effect associations did not depend on the action control mode in which they were learned. Only the *use* (operationalized in the test phase) seems to be dependent on the action control mode.

As illustrated above, the acquisition and use of action-effect binding under different action control modes have been primarily studied with rather simple choice-reaction tasks in free- and forced-choice designs. Although action-effects are assumed to play a crucial role in response selection, there are only a few studies targeting the impact of action-effects in task selection. Task selection is often studied with the task-switching paradigm. Task-switching reflects the flexibility of the cognitive system when being confronted with multiple task requirements. In everyday life, we often have to decide what to do. Thus, we perform an action in order to achieve a goal by neglecting all the other opportunities that could interrupt the ongoing action. But if a new goal or task is more prominent, the cognitive system must be able to abandon the current task by reconfiguring the current task set in order to select and perform another action.

According to Logan and Gordon (2001) and Logan and Schneider (2010) a task-set can be defined as a set of parameters that program task-specific processes such as perceptual encoding, memory retrieval, response selection, and response execution. If action-effects influence response selection as seen in experiments with free- and forced choice designs, it is conceivable that they will also influence task selection and task execution in task-switching. In a study by Kiesel and Hoffmann (2004), pressing a key (one and the same action) led to two different action effects (short/fast vs. long/slow movements of the target) in a horizontal and vertical arrangement: Reactions were slower in the slow-movement context and faster in the fast-movement context, although the target movements occurred *after* the response was given. Thus action-effect associations are acquired context specifically and the context influences the way the same action (pressing a key) is performed (slowly or quickly). In a study carried out by Ruge et al. (2010), two target stimuli were horizontally and vertically aligned. A cue indicated whether participants had to determine the position of the horizontal or the position of the vertical stimulus. Two different effect modes followed responses. In the task-related effect condition, a red square appeared in the position of the correct response (e.g., left in the horizontal condition or above in the vertical condition). In the task-unspecific effect condition, participants were just told whether they had performed correctly or not. The authors found a significant two-way interaction between task transition and effect type for trials with a long-cued target interval (CTI, i.e., 1500 ms): in the task specific feedback condition, switch costs were reduced. The authors interpret this result as meaning that task-specific feedback can help to disambiguate task-ambiguous response meanings (that is, the same response for two different tasks).

In order to further study the role of action-effects in task-switching, Lukas et al. (2013) devised a new paradigm. In an acquisition phase, participants performed magnitude and parity tasks in a cued task-switching paradigm. A cue presented to the target (a number between one and nine without the five) indicated which task to perform. Correct responses were immediately followed by consistently occurring action-effects in the experimental group and by inconsistent, random action effects in the control group. In the transfer phase, the consistently and regularly occurring action-effects changed to a random mapping, so that the learned action-effect associations were no longer valid. If action-effect associations were anticipated and facilitated, implying discrimination between competing task sets, then switch costs should be lower in the experimental group in the acquisition phase and should increase in the transfer phase. This is exactly what Lukas et al. (2013) found – at least for trials with a short cue target interval (CTI). They interpreted the reduced switch costs in the acquisition phase to be due to the fact that participants activate the action effects as part of the current task set. This helps to differentiate competing task sets. However, in trials with long CTIs, the task set is already fully prepared so that there is no additional benefit by consistently occurring task effects. In the test phase, it was shown that switch costs were increased after the learned action-effect associations were no longer valid. This is further evidence that effects that occur as a *consequence* of an action play an important role, not only in simple-choice designs but also in more complex task designs. Recently, this effect was replicated not only for switch costs, but also for *N* – 2 repetition costs (Schuch et al., 2017). Moreover, it is noteworthy that acquisition as well as the use of action-effect associations could be shown in a design that is comparable to a forced-choice design (neither the task nor the key could be freely chosen by the participants). Herwig and Waszak (2009) already assumed that with more complex S-R mappings, action effects might become more important and hence participants rely more on action-effect associations.

To pursue this thought, we conducted the present study with a cued task-switching paradigm (forced-choice design) and a voluntary task-switching paradigm. Although key strokes in a voluntary task-switching paradigm are not completely free-choice (there is a correct and a wrong response), participants still have the freedom to choose the task they want to perform. Hence, we equalize this paradigm with free-choice designs in simple-response studies. In line with the results obtained by Lukas et al. (2013) and also at least tending in Schuch et al. (2017) with respect to *N* – 2 repetition costs, we assumed that consistent action-effect mappings in the acquisition phase should lead to better performance than randomly assigned action-effects in both task-switching paradigms. However, the main focus of the present study was to investigate *consistency effect* (i.e., tasks are chosen according to previously learned task-effect associations) and *non-reversal advantage* (i.e., previously learned task-effect associations improve task performance and switching between tasks when matching effects are presented before task selection) by employing a task-switching paradigm. Thus, we introduced a cued task-switching paradigm, similar to the forced choice designs (sensorimotor learning mode) and a voluntary task-switching paradigm similar to the free-choice designs (ideomotor learning mode). In two experiments, participants learned task-response-effect associations either in a cued or in a voluntary task-switching design. In Experiment 1, consistency was tested by presenting the previous learned action-effects before task selection in a voluntary task switching paradigm (to distinguish the preceding “action”-effects better from the action effects in the acquisition phase, they are called *practiced effects* in the following). In line with consistency effect, subjects should tend to choose the task that was previously followed by the respective effect. In Experiment 2, the non-reversal advantage was tested by presenting the previous learned action-effects before the task cue. In line with non-reversal advantage, subjects should react faster and be less error prone when the respective practiced effect matches the following task. Moreover, we were interested in determining whether the integration of action effects in a task set is limited to the response level or takes place on a higher hierarchical task level. That means, for instance, that participants have learned the association between pressing a certain key and the screen turning to green for numbers smaller than five. When, during the test phase, the practiced effect is a screen turning to green and the target number is *greater* than five, they are more likely to press the key assigned in which the screen turns to yellow when task-effects are integrated on the task level.

## Experiment 1

### Materials and Methods

#### Participants

Eighty participants (59 female, 21 male) took part in Experiment 1 (age range 18–29, *M* = 21.4, *SD* = 2.3). The subjects were randomly assigned to experimental and control groups in both learning modes (ideomotor learning – voluntary task-switching vs. sensorimotor learning – cued task-switching). The experimental groups received consistent, predictable action-effects, whereas the control groups received random, non-predictable action-effects. Hence, in the control groups, no action-effect learning could take place (see paragraph Stimuli, tasks and action effects for further explanation).

Subjects were undergraduates who either received partial course credit or a monetary reward of 10 € each. Ethical approval was not required for this study in accordance with national and institutional requirements. All procedures performed in this study were in accordance with the ethical standards of the 1964 Helsinki declaration and its later amendments. Informed consent was obtained from all individual participants.

#### Stimuli, Tasks, and Action Effects

The experiment was programmed in PsychoPy, v1.83.01 (s. Peirce, 2007, 2009) and ran on a Baron Shuttle PC (CPU 3.5 GHz). Stimuli were presented on a Dell monitor with a display diagonal of 22″. Participants sat in front of the screen at a viewing distance of approximately 60 cm. The stimuli consisted of digits ranging from one to nine without a five. They appeared in white on a black background in the center of the screen with a height of 1.3 cm (visual vertical angle 1.24°). Participants had either to decide whether a number was larger or smaller than five (magnitude task) or whether it was odd or even (parity task). For one task, they had to press the period-key and the y-key with the right and left index finger, for the other task they had to press the q-key and the p-key with the right and left middle finger. The keys arranged on the left side of a standard QWERTZ-keyboard were always assigned to odd or less than five, and the keys arranged on the right side were assigned to even or greater than five. The task-key assignment was counterbalanced across participants.

Participants of the ideomotor learning groups (*EG 1* and *CG 1*) were instructed to choose between one of two tasks based on these instructions: “In this experiment you have to perform one of two tasks, the magnitude task or the parity task [...]. You yourself may choose which task you are going to perform next. Keep in mind that you must switch regularly, so that you are performing the two tasks in an approximately equal proportion.”

**FIGURE 1:**
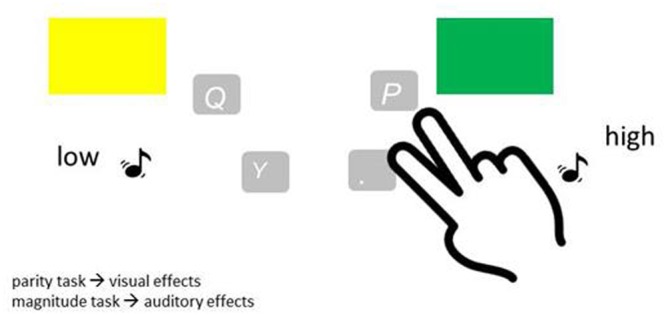
Example of a task-effect assignment for the experimental groups. Performing one task (here: the parity task, which was executed with the response keys q and p) elicited visual action effects, the respective other task (here: the magnitude task, executed with the response keys y and period) auditory action effects.

In the sensorimotor learning groups (*EG 2* and *CG 2*), participants were cued as to which task to perform. The cues were a square or a diamond that framed the stimulus. The square indicated the parity task, the diamond the magnitude task. First, a fixation cross appeared in the middle of the screen for 500 ms. Immediately after, the cue was presented and 500 ms later the stimulus appeared (CTI = 500 ms). Cue and stimulus stayed on the screen until a response was obtained. If there was no response after 2500 ms, they disappeared and a message appeared, prompting participants to respond faster. If participants pressed the wrong key, an error message appeared. The error message was displayed on the screen for 500 ms immediately after the wrong response with a letter height of 0.3 cm (during the acquisition phase and the relearn block). In the test phase, errors were indicated on the screen with a more prominent letter height of 1.3 cm. Only correct responses were immediately followed by action-effects. For one task, a large green or yellow square (19.5 cm in length) appeared as action effect on the screen for 500 ms and a high or a low-pitched tone was emitted for 500 ms for the other task. On the response level, the action effects differed in regard to their features (green, yellow, high or low). The action-effects differed also on a higher dimensional level, i.e., for one task visual action effects appeared and for the other task auditory action effects appeared. The assignment of tasks and action-effects (e.g., visual/magnitude task, auditory/parity task) was counterbalanced across participants (see for example **Figure 1**). The green square and the deep tone were always assigned to the responses “even” or “smaller.” The yellow square and the high tone were always assigned to the responses “odd” or “larger,” respectively. In the control groups (*CG* 1 and *CG* 2), action-effects were randomly assigned. Each response-effect combination was possible with equal probability. Therefore, there was no consistency between response and effects, neither on the response level nor on the task level.

In the test phase of both experiments, the practiced effects were presented before task selection. Every practiced effect could now be followed by any target. That is, 32 combinations of practiced effect and target were possible, of which half of them were learned associations and half of them had not been associated before. For instance, the low tone was associated with the response “smaller” and hence with small target numbers, but never with larger target numbers. A low tone followed by the number 9 would hence be an unknown association. The practiced effect (presented for 500 ms) was followed by the target after a gap of 200 ms (i.e., inter-stimulus interval [ISI] = 700 ms). Participants were instructed to freely choose which task to perform. After correct responses, the screen stayed black for 900 ms. If the response was wrong, an error message appeared on the screen for 500 ms. The screen turned black for a further 400 ms until the next trial began. After each block, participants received feedback concerning their mean response time and the amount of correctly executed tasks. They were reminded to respond as quickly and correctly as possible.

#### Procedure and Experimental Design

Participants were instructed in written form and additionally orally if further explanation was needed. The experimental group was not explicitly informed about the action-effect association. Both groups were told that they could use the action-effects as feedback if the task was performed correctly, because after a wrong response, no action-effect occurred. Moreover, they were asked to respond as quickly and as correctly as possible.

**FIGURE 2:**
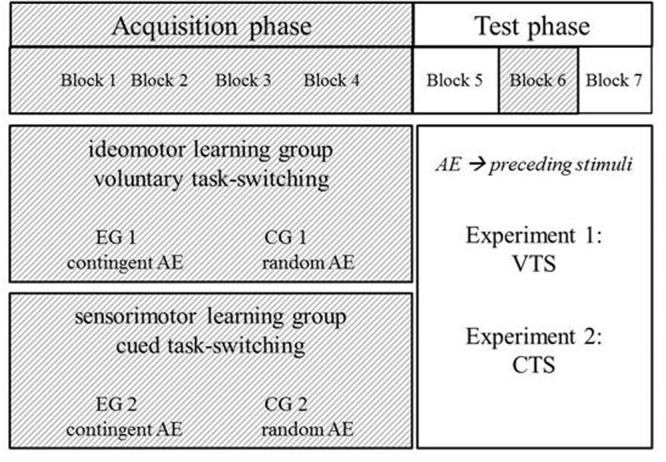
Experimental design for both experiments: *CG* 1 and *CG* 2 – control groups, *EG* 1 and *EG* 2 – experimental groups. 1 stands for voluntary task-switching (VTS), 2 for cued task-switching (CTS), AE for action effects. In Experiment 1, the test phase was voluntary task switching, in Experiment 2, the test phase was cued task switching. In the test phase, action effects became preceding effects (practiced effects).

The session started with a short practice block consisting of 16 trials, which were not registered. The experiment consisted of seven blocks of 64 trials: five acquisition blocks, in which the subjects learned action-effect associations and two test blocks, in which the effect of the learned associations was tested. After the fourth acquisition block, the first test block was conducted (Block 5). Subsequently, the fifth acquisition block (Block 6) was presented, functioning as a relearn block, and serving as an update for learned action-effect associations (see **Figure 2**). Finally, the second (and last) test block (Block 7) was performed.

Several analyses were conducted to test different hypotheses. In Experiment 1, the focus was on the consistency effect. For that reason, the task-choice ratio for consistent tasks was tested in the test phase as a dependent variable. A chosen task was defined as consistent when it matched the practiced effect according to the previously learned action-effect association. That is, the task followed by a visual effect in the acquisition phase was also chosen when a visual effect preceded the task stimulus. Condition (experimental vs. control) and learning mode (voluntary task-switching vs. cued task-switching) were between-subject independent variables. For performance measurements, RT and error rate were dependent variables, and task transition (repetition vs. switch) and block (acquisition blocks vs. test blocks) were independent variables.

### Results

#### Consistency Effect

To analyze the consistency effect, first, the amount of task-consistent vs. inconsistent task choices was enumerated. Although no consistent response-effect associations could be seen in the control groups, their responses were categorized as “task-consistent and task-inconsistent” in the same way as the responses of the parallelized experimental groups. That is, if the experimental group had experienced that the response “even” elicited a green square, choosing the parity task (by answering with the odd or even key) was considered as task consistent. In the same way, also the task choice of the control group was categorized (choosing the parity task after a green square is “task consistent”), although the control group had no association between a green square and the response “even.”

Participants in the voluntary task switching blocks who performed less than five switches or repetitions in the acquisition phase or performed one task only more than 54 times in either in an acquisition block or in a test block (comprising 64 trials) were excluded from analyses. 34 (of 80) participants met these criteria. In the voluntary task switching group, 8 control and 13 experimental condition participants had to be excluded. In the cued task switching group, 5 control and 8 experimental condition participants had to be excluded. The distribution of remaining participants in each condition is shown in **Table 1**.

**Table 1:** Distribution of participants in Experiment 1 in each condition after selection.

Condition in the acquisition phase	Participants
Voluntary task switching, experimental	10
Voluntary task switching, control	9
Cued task switching, experimental	13
Cued task switching, control	12

Trials in which errors were committed and trials following these were also excluded. On average, 16.2% of the trials were erroneously performed. A two-way ANOVA with the independent between-subject variables condition (experimental vs. control) and learning mode (voluntary task switching vs. cued task switching) and the dependent variable task-choice ratio of consistent tasks (in percent) was conducted.

The main effect condition only tended to be significant, *F*(1,40) = 3.18, *p* = 0.08, $\eta_{p}^{2}$ = 0.07. Participants in the experimental groups made task-consistent task choices in 54.5% (*SE* = 2.6). Participants in the control groups made task-consistent task choices in 47.7% (*SE* = 2.8). Task consistent choice ratio did not differ significantly from 50% in both groups (*t*[22] = 1.4, *p* > 0.05 for the experimental groups and *t*[20] = -2.0, *p* > 0.05 for the control group). Neither the main effect learning mode, *F* < 1, nor the interaction between condition and learning mode were significant, *F* < 1.

In order to take a closer look at the consistency effect, we conducted two additional two-way ANOVAs. In a voluntary task-switching paradigm, comprising two tasks, participants were allowed to choose between two correct responses. In the test phase, that means that the responses can be task consistent or task inconsistent with respect to the practiced effects. Moreover, task consistent responses can be response consistent or response inconsistent. For instance, a green square was associated with the response “even” in the acquisition phase. In the test phase, however, it was possible for a green square to be followed by the number 7. These two stimuli have never been associated before because 7 is an odd number. However, if the participant still chose to perform the parity task, this trial was task consistent, but response inconsistent. Trials in which the practiced effect and a possible correct response match the formerly learned action-effect associations are both task consistent and response consistent. Task and response consistent trials were analyzed separately from other trials. The expected resulting pattern from choosing responses consistent with the former learned action-effect associations would suggest that task-effects are integrated in a task set on the response level. Analyzing the other trials, in which neither of the correct responses matched the learned response-effect associations, should provide evidence as to whether action-effects are also integrated on a higher task level. Although not matching on the response level, the response in which the associated action-effect shares the same modality as the practiced effect fits on the task-level. We assumed that pressing these keys in a non-random manner may show that task-effects are not just associated to the motor response patterns for pressing a key, but also integrated into the mental representation of the numerical categorization.

#### Analysis on the Response Level

In those trials in which correct responses matched formerly learned action-effect associations, participants of the experimental groups made response consistent choices in 27.9% (*SE* = 1.3) of the trials (please note that only 25% of all trials provide the possibility to be task compatible as well as response compatible). The control groups chose the matching response in 23.8% (*SE* = 1.4) of the trials. The difference in task-choice ratio was reflected by a main effect condition, *F*(1,40) = 4.6, *p* < 0.05, $\eta_{p}^{2}$ = 0.1. Neither the main effect learning mode, nor the interaction between condition and learning mode was significant, *F*s *<* 1.

#### Analysis on Task Level

In those trials in which neither of the correct responses matched formerly learned action-effect associations on the response level, participants of the experimental groups made task consistent (but response inconsistent) choices in 26.6% (*SE* = 1.5) of the trials (please note, that like above, only 25% of all trials provide the possibility to be task compatible, but response incompatible). The control groups chose this response-effect pattern 23.9% (*SE* = 1.6) of the time. This difference was not significant. Neither the main effect condition, nor the main effect learning mode, nor the interaction between condition and learning mode were significant, *F*s < 1.6.

Since by means of standard null-hypothesis testing the non-existence of an effect may not be confirmed, we additionally applied a Bayesian alternative developed by Wagenmakers (2007) as suggested by Masson (2011). The BIC, an index commonly used to quantify goodness-of fit of a formal data model, is applied for generating an estimate of the Bayes factor, $\mathrm{BF}\approx \frac{p\mathrm{BIC}({D|H}_{0})}{p\mathrm{BIC}({D|H}_{1})}=e^{\left(\Delta \mathrm{BIC}\right)/2}$. The calculation yielded a Bayesian factor of *BF* = 3.0.

The posterior probability favoring the null-hypothesis, that there is no effect condition on task-choice ratio, was $p_{\mathrm{BIC}}(H_{0}|D)\;=\;\frac{\mathrm{BF}}{BF+1}\;=\;75\%$. The subsequent probability, favoring the alternative hypothesis, that participants in the experimental groups would make more consistent task-choices than that of the control groups, was p_BIC_ (H_1_|D) = 1 - p_BIC_ (H_0_|D) = 25%.

To provide comparability to the *BF* on the task level, also the *BF* on the response level was calculated and yielded a *BF* = 0.7. The posterior probability favoring the null-hypothesis that there is no effect on the response level was p_BIC_ (H_0_|D) = 42%. Consequently, the posterior probability favoring the alternative hypothesis was p_BIC_ (H_1_|D) = 58%.

### Discussion of Experiment 1

The results of Experiment 1 showed indeed that participants in the experimental groups favored tasks that were previously associated with the stimulus that now preceded the task choice. However, this was only significant on the response level. That is, only when the practiced effect and the target had been associated before and hence allowed a previously associated response, was it possible to see a choice in favor of the matching task. If practiced effect and target had not been previously associated, it was not possible to see a choice in favor of the task that was associated with the practiced effect. Hence there is doubt as to whether real task-effect associations do occur. Our results at least indicate a stimulus-response-effect association on a lower hierarchical level. However, one can also not state that no real task-effect associations exist. The Bayesian factor only shows weak evidence for favoring the null-hypothesis. Due to the large amount of subjects that had to be excluded, we lost test power in no small measure. Therefore one can also argue that the effect was too small to be detected by the remaining sample size.

## Experiment 2

### Materials and Methods

#### Participants

Seventy-five participants (50 female, 25 male) took part in Experiment 2 (age range 18–37, *M* = 20.6, *SD* = 2.3). As in Experiment 1, the sample consisted of undergraduates who either received partial course credit or a monetary reward of 10 €.

#### Stimuli, Tasks, and Action Effects

Stimuli, tasks and action effects were designed in a similar way to Experiment 1. The acquisition phase was exactly the same as in Experiment 1. The difference between Experiment 1 and Experiment 2 was the test phase. In Experiment 1, we focused on the consistency effect. In Experiment 2, attention was focused on the non-reversal advantage. For this reason, in the test phase, the formerly learned action effects turned to preceding practiced effects with a cued task-switching design. The practiced effect was presented for 500 ms. After the practiced effect disappeared, 200 ms later the task cue and the target were simultaneously presented. As in Experiment 1, the cues were a square or a diamond that framed the stimulus.

#### Procedure and Experimental Design

The procedure was the same as in Experiment 1. However, the analyses differed, as the focus was on the non-reversal advantage. Data from the test phase (Block 5 and Block 7) of Experiment 2 were analyzed using a three-way ANOVA with the between subject variables condition (control vs. experimental), learning mode (voluntary vs. cued task-switching) and task consistency (task-consistent vs. task-inconsistent). Dependent variables were RT and error rate.

## Results

### Non-reversal Advantage

Like in Experiment 1, participants in the voluntary task switching blocks who performed less than five switches or repetitions in the acquisition phase were excluded from analyses. Participants who did not show any (correct) switch trials in two or more blocks of the acquisition phase or who failed to switch correctly in one or both test blocks were excluded from analyses due to not following instructions. 18 participants of 75 were excluded from analyses, all of them were in the ideomotor learning group, 9 in the experimental and 9 in the control group (see **Table 2** for distribution in each condition for the remaining participants). For RT analysis, trials in which errors were committed and trials following these were also excluded. Furthermore, all trials exceeding three standard deviations above the mean of RT and trials with an RT of less than 200 ms were omitted.

**Table 2:** Distribution of participants in Experiment 2 in each condition after selection.

Condition in the acquisition phase	Participants
Voluntary task switching, experimental	12
Voluntary task switching, control	10
Cued task switching, experimental	18
Cued task switching, control	18

#### RT

Mean values in every condition and SE are shown in **Table 3**. None of the main effects reached significance, *F*s < 1. Likewise, none of the two-way interactions reached significance, *F*s < 1. The three-way interaction of condition, learning mode and task consistency, however, tended at least to be significant, *F*(1,54) = 3.1, *p =* 0.08, $\eta_{p}^{2}$ = 0.05. Numerically, participants who had performed voluntary task switching in the acquisition phase were faster in task consistent trials than in task inconsistent trials. The *BF* of the three-way interaction is 1.6, resulting in a p_BIC_ (H_0_|D) = 62% and a p_BIC_ (H_1_|D) = 38%.

**Table 3:** Mean RT (and SE) in ms in the test phase as a function of condition, learning mode and task consistency.

			Task consistency	
		Learning mode	Task consistent	Task inconsistent
Condition	Experimental	Voluntary task switching	918 (54)	956 (55)
		Cued task switching	1056 (44)	1056 (45)
	Control	Voluntary task switching	933 (59)	902 (60)
		Cued task switching	1020 (44)	1047 (45)

#### Error Rate

**FIGURE 3:**
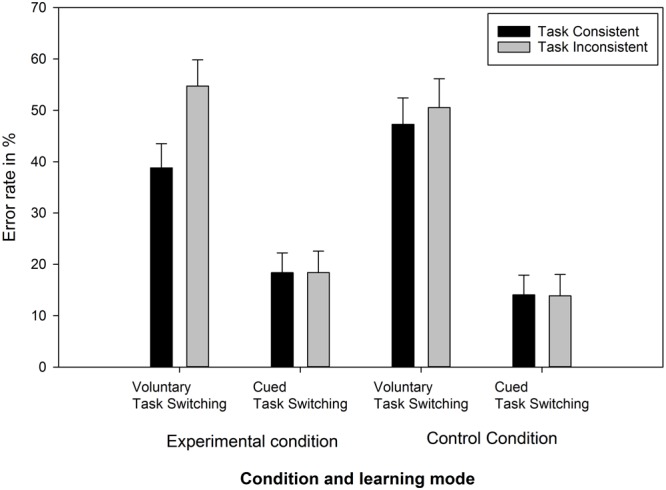
Error rate in percent of Experiment 2 in the test phase as a function of experimental condition, learning mode in the acquisition phase and task consistency.

Error rate data were first arcsine transformed, before being entered into the three-way ANOVA with the variables condition, learning mode, and task consistency (see **Figure 3**). The main effect of task consistency was significant, *F*(1,54) = 6.98, *p* < 0.05, $\eta_{p}^{2}$ = 0.11. Task consistent trials yielded fewer errors (29.6%) than task inconsistent trials (34.4%). The main effect of learning mode was also significant, *F*(1,54) = 54.0, *p* < 0.001, $\eta_{p}^{2}$ = 0.5. Participants, who had performed voluntary task switching in the acquisition phase, showed a higher error rate (47.8%) compared to participants who performed cued task switching (16.2%). Likewise, the interaction of task consistency and learning mode was significant, *F*(1,54) = 8.25, *p* < 0.01, $\eta_{p}^{2}$ = 0.13. Only in the voluntary task switching learning mode, task consistent trials yielded fewer errors than task inconsistent trials (43.0% vs. 52.6%), in the cued task switching learning mode, the error rates were the same (16.2% vs. 16.1%). The two-way interaction of task consistency and condition just failed significance, *F*(1,54) = 3.9, *p* = 0.052, $\eta_{p}^{2}$ = 0.07. In the experimental groups, the error rate was reduced to a higher amount for task-consistent trials (28.6% vs. 36.6%) compared to the control group (30.7% vs. 32.2%). The main effect of condition was not significant (*F* < 1). The three-way interaction of condition, learning mode and task consistency only tended to be significant, *F*(1,54) = 3.0, *p* = 0.09, $\eta_{p}^{2}$ = 00.05. The *BF* of this three-way interaction was 1.7, p_BIC_ (H_0_|D) = 63% and a p_BIC_ (H_1_|D) = 37%.

### Reaction Time and Error Rates in the Acquisition Phase

Since the acquisition phase and the relearning block of both experiments were the same, data from both experiments were merged to analyze whether consistent action-effects in a voluntary and cued task-switching paradigm would reduce performance costs. For reaction time analysis error trials and trials following an error trial were excluded from analyses. All trials that exceeded three standard deviations of the mean RT or had a RT of less than 200 ms were omitted. Moreover, the same participants that had been excluded from analysis in Experiment 1 and Experiment 2 were excluded.

#### RT

A three-way ANOVA with the variables condition, learning mode and task transition revealed a main effect of condition, *F*(1,98) = 4.7, *p* < 0.05, $\eta_{p}^{2}$ = 0.05. Participants in the experimental groups were faster (704 ms) than participants in the control groups (758 ms). The main effect of task transition was also significant, *F*(1,98) = 68.7, *p* < 0.001, $\eta_{p}^{2}$ = 0.41. Participants reacted slower in switch trials (769 ms) compared to repetition trials (692 ms). The two-way interaction of condition and task transition was also significant, *F*(1,98) = 4.8, *p* < 0.05, $\eta_{p}^{2}$ = 00.05. Switch costs were higher in the experimental conditions (97 ms) than in the control conditions (57 ms). Likewise, the two-way interaction of learning mode and task transition was significant, *F*(1,98) = 5.3, *p* < 0.05, $\eta_{p}^{2}$ = 0.05. Switch costs were lower in the voluntary task switching mode (55 ms) than in the cued task switching mode (98 ms). There was also a two-way interaction of condition and learning mode, *F*(1,98) = 8.0, *p* < 0.01, $\eta_{p}^{2}$ = 0.07. With consistent action-effects (experimental condition), participants performing voluntary task-switching were faster (676 ms) than participants with random action effects (control condition; 799 ms). Participants performing cued task-switching showed similar RTs in the experimental and control condition (732 ms vs. 716 ms). The main effect learning control mode was not significant, *F* < 1, neither was the three-way interaction between condition, learning mode and task transition, *F* < 1.

#### Error Rate

Concerning the error rate, first the data were arcsine transformed and then the same three-way ANOVA as for the RT was applied. The main effect of condition was significant, *F*(1,98) = 4.7, *p* < 0.05, $\eta_{p}^{2}$ = 0.05. Participants receiving consistent action effects yielded fewer errors (10.4%) than participants receiving random action effects (14.6%). Like for the RT, also the main effect of task transition was significant, *F*(1,98) = 31.2, *p* < 0.01, $\eta_{p}^{2}$ = 0.24. Participants made fewer errors in repetition trials (11%) than in switch trials (14%). The two-way interaction of condition and learning mode was significant, too, *F*(1,98) = 15.7, *p* < 0.01, $\eta_{p}^{2}$ = 0.14. Participants in the voluntary task switching mode were less error prone in the experimental condition than in the control condition (6.9% vs. 19.2%). In contrast, participants in the cued task switching mode showed numerically less errors in the control condition (10%) than in the experimental condition (14%). No other main effect or interaction was significant, *F*s < 1.

### Discussion of Experiment 2

Concerning RT, the non-reversal advantage could not be shown, but it was seen in the error rates. Task consistent trials yielded fewer errors than task inconsistent trials. This effect was qualified by the two-way condition of task consistency and learning mode. Only in the voluntary task switching learning mode, task consistent trials yielded fewer errors than task inconsistent trials (43.0% vs. 52.6%). In the cued task switching learning mode, the error rates did not differ (16.2% vs. 16.1%). It is also seen that the error rates of the voluntary task switching mode was much higher in the (cued) test phase than of the cued task switching learning mode. This effect was only seen in the two test phases. Hence it cannot be traced back to a general error in the experimental procedure, nor to a lack of motivation of the participants. It seems that the shift from a voluntary task switching design (in which two of four task keys were correct) to a cued task-switching design (in which only one of four task keys is correct) causes strong confusion, requiring high cognitive effort and leading to a high error rate. Although there are several studies that combine free-choice tasks with forced-choice tasks (e.g., Fröber and Dreisbach, 2017; Naefgen et al., 2017), those different tasks were rather intermixed and participants were aware of both task types. In our case, those participants who were trained in a voluntary task-switching paradigm did not know they would have to shift to a cued task-switching paradigm. Whether this unawareness alone causes the high amount of errors or whether the required high cognitive effort to suppress formerly learned correct responses as wrong responses alone leads to this effect cannot be answered in this study and requires further research.

Analyses of RT and error rate revealed a general performance improvement with consistent action effects compared to random action effects. This effect was only seen in the voluntary task switching mode. Participants were fastest in the voluntary task-switching design. This is noteworthy, as usually free-choice tasks are executed more slowly than forced-choice tasks (e.g., Naefgen et al., 2017). This usual pattern was also seen in the control conditions. Action effects seem to have a general facilitating effect in voluntary task switching. This facilitating effect was mainly seen in task repetition trials. This is why in the present study, switch costs were affected only negatively, at least concerning RT. Hence, we failed to replicate findings in which action effects helped specifically to distinguish task set and led to decreased switch costs (e.g., Ruge et al., 2010; Lukas et al., 2013).

## General Discussion

In the current study, we targeted whether action-effects are involved in task selection and execution under different action-control modes (sensorimotor vs. ideomotor). For this reason, three measurements were analyzed: consistency effect, which reflects whether bi-directional learning also occurs in task selection, non-reversal advantage, which shows if task execution is facilitated when combined with task-consistent practiced effects, and task performance (RT, error rate and switch costs) when action effects are consistent compared to when they are completely random. Participants learned stable action-effect associations in a task-switching paradigm in the experimental groups, while the control groups performed completely random action-effects that could not be anticipated. Two action-control modes were used in those acquisition phases: a voluntary task-switching design, in which participants followed an ideomotor learning mode, and a cued task-switching design, in which participants followed a sensorimotor learning mode.

### Consistency Effect

In Experiment 1, the previously learned action effects turned to practiced effects in the test phase. The test phase consisted of a voluntary task-switching paradigm. The ratio of the chosen tasks that matched the previously learned task-effect association was analyzed (i.e., consistency effect). It was shown that participants chose responses matching formerly learned response-effect mappings in greater number than those due to random effects. This was independent of the learning mode. That is, we can confirm that the acquisition of action-effect associations takes place in a more complex task environment in sensorimotor as well as in ideomotor learning modes (cf. Pfister et al., 2011).

By splitting up the data in the test phase of Experiment 1, we found that participants selected responses that matched previously learned stimulus-response-effect-associations above random response rates. However, participants did not select tasks according to task-effect associations. Sharing the same modality did not seem to be sufficient to link to one task (i.e., visual effects are linked to the parity task). Accordingly, the integration of sensory effects in the task-set of the numerical selection tasks, applied in this study, seems to take place on a response level and thus prompts the key-press (the goal-directed movement) and not the mental categorization *per se*. This does not mean that task-effect associations do not exist. However, in this study they were too weak to show evidence for bi-directional task-effect associations. It is for instance conceivable that task effects need to be task-relevant or that tasks and effects have a logic relation to one another. Hence, the mechanisms underpinning the retrieval process are still not clear. Referring to feature-integration theory, the preceding stimuli trigger the response only on condition that there is a complete feature overlap. If under the same condition (e.g., number less than five), there is a feature-mismatch (the color of the preceding stimulus is yellow and not green as in the acquisition phase), the response belonging to the matching task (magnitude) is not prioritized. Further research is necessary in order to determine whether the retrieval mechanisms of task-effects work in an all-or-none manner or rather in a manner of graded/weighted correspondence/overlap.

### Non-reversal Advantage

In Experiment 2, the non-reversal advantage was the focus of interest. The acquisition phase was exactly as in Experiment 1, but the test phase consisted of a cued task-switching paradigm. The cued task was preceded by formerly learned action effects. We analyzed whether task performance was facilitated when the preceding effects matched formerly learned action-effect associations. Regarding RT, there was only a small hint with a tendency that participants who had acquired action-effect associations in an ideomotor learning mode reacted faster in task consistent trials. The results of the error rate support this tendency. Task-consistent trials were less error prone than task inconsistent trials only in the ideomotor learning groups and rather for the experimental groups. There are several explanations as to why the effect of non-reversal advantage was not clearer. The main problem is, as mentioned above, the loss of power due to much exclusion of participants from analyses. Moreover, with respect to the non-reversal advantage one has to keep in mind that in earlier studies (e.g., Elsner and Hommel, 2001; Herwig and Waszak, 2009) the preceding effect itself served as an imperative stimulus and no additional cue was presented. It is possible that the cue in the cued task-switching paradigm overlaps the non-reversal advantage. Maybe the non-reversal advantage would be more prominent if the practiced effect itself would serve as cue.

Also the CTI has to be taken in consideration with respect to the results. In the Lukas et al. (2013) study, two different CTIs had been applied. The worsening effect of task-switch costs when changing consistent action effects into random action effects was only seen in the condition with short CTI. Schuch et al. (2017) used a CTI of 500 ms. They found clear effect of increased switch costs only in the error rate. In the present study, the CTI was also 500 ms in the acquisition phase and even longer (700 ms) in the test phase. It is possible that the effect of action effects in task switching is transient and other cognitive processes take over control in task performance with longer CTI.

### Task Performance

Analyses of the acquisition phase revealed that action effects especially help task performance in a voluntary task-switching paradigm: RT is faster with consistent action effects during voluntary task-switching. Usually, free-choice tasks are performed slower than forced-choice tasks, as they are accompanied by additional cognitive processes. It is assumed that these processes reflect generating internally a task goal (see Naefgen et al., 2017). Providing action effects in a voluntary task-switching paradigm might accelerate this goal generating process.

Previous studies have shown that action effects can help to create task-ensembles (Weaver and Arrington, 2013). Although no task-ensembles were used in the presented study, but only a flat non-hierarchical task structure (to each task and each response belonged one specific effect), it was assumed that action effects in a voluntary task-switching paradigm can contribute in reducing switch costs, as has been shown by previous studies with a forced-choice task switching paradigm (Ruge et al., 2010; Lukas et al., 2013). However, we found larger switch costs for the groups with consistent action effects than for the groups with inconsistent action effects. Likewise, in the study of Lukas et al. (2013), the comparison took place between “predictable action effects” and “random, non-predictable action effects.” In the study of Ruge et al. (2010) the comparison took place between “task-related effect feedback” and “non-specific accuracy feedback.” But in both studies, a forced-choice task-switching paradigm was conducted. Hence, one could assume that in a voluntary task-switching paradigm, action effects are not helpful to reduce switch costs. This assumption, however, is in contrast to the assumption that action effects are especially effective in an ideomotor action control mode (e.g., Pfister et al., 2010, 2011; Herwig and Waszak, 2012). In the present study, action effects seemed to be especially efficient to reduce RT of repetition trials, hence increasing switch costs. Further research is needed to investigate under which conditions action effects can reduce switch costs by also reducing the RT of the switch trials.

The absent effect of reduced switch costs, however, does not lessen the effect action effects have on task selection. Also Arrington and Yates (2009) and Arrington et al. (2010) proposed that task selection and task performance are independent of each other. The differentiated effects only provide further evidence for this assumption.

## Conclusion

The results of our study broaden this previous research by applying and combining two different task-switching paradigms with action effects: a cued task-switching paradigm and a voluntary task-switching paradigm. In previous research it was shown that action effect associations of simple response-effect associations were learned in both learning modes: ideomotor and sensorimotor control (Pfister et al., 2011), but evidence for the use was only seen in the ideomotor control mode. Accordingly, we find that the consistency effect was found independently of how action-effect associations were learned, but only on the response level. That is, only when the practiced effect and possible responses had been associated before, the matching response is selected. Concerning the non-reversal advantage, we find support for the idea that the mode in which action-effect association are learned in more complex environments affect task performance differently. Whether this means that two different action control systems underlie this effect cannot be answered with this study. Janczyk et al. (2015b) for instance argue against the separation of two action control systems. Although we cannot reject this statement based on our results, the debate of how learning mode affects task performance is not yet completed.

Janczyk et al. (2015a) question whether free-choice tasks are an appropriate method to study voluntary actions. One might also raise the question with respect to our study. We argue that it was exactly the underlying task (and the accompanying higher cognitive effort) that we wanted to investigate with respect to action-effect learning. Although we are not convinced that it is completely inappropriate to study action control with voluntary responses, we concede that one might consider alternative research methods in the future.

To sum up, it was shown that specific action-effect association were used for task selection in more complex task environments. Evidence for non-reversal advantage was rather shown for error rate. Action effects help to reduce reaction time in a voluntary task switching paradigm, but switch costs are not affected.

## Author Contributions

AS and SL designed the study. AS executed data collection. SL analyzed the data. AS wrote a first draft of the manuscript, SL revised it.

## Conflict of Interest Statement

The authors declare that the research was conducted in the absence of any commercial or financial relationships that could be construed as a potential conflict of interest.
